# Commentary: Methods for calculating growth trajectories and constructing growth centiles

**DOI:** 10.1002/sim.8129

**Published:** 2019-07-12

**Authors:** T. J. Cole

**Affiliations:** ^1^ UCL Great Ormond Street Institute of Child Health London UK

## Abstract

This commentary rounds off a collection of papers focusing on statistical methods for analysing growth data. In two papers, Anderson and colleagues discuss growth trajectory models in early life, using data on height and weight from the HBGDki initiative, while two papers from Ohuma and Altman review methods for centile construction, with data from the INTERGROWTH‐21^st^ project used to provide worked examples of centiles for birthweight and fetal head circumference. Anderson et al focus on four growth trajectory models: quadratic Laird‐Ware, SITAR, brokenstick, and FACE, where the latter two fit better than the former two applied to length data in individuals. On this basis, they recommend brokenstick and FACE for future work. However, they do not discuss the timescale on which the growth models assess growth faltering nor the relevance of this timescale to later health outcome. Models that best detect short‐term fluctuations in growth (brokenstick and FACE) may not necessarily be best at predicting later outcome. It is premature to exclude the quadratic Laird‐Ware or SITAR models, which give a parsimonious summary of growth in individuals over a longer timescale. Ohuma and Altman highlight the poor quality of reporting in fetal centile studies, and they provide recommendations for good practice. Their birthweight centiles example illustrates both the power of the GAMLSS software and its capacity for misuse. The longitudinal fetal head circumference centiles are biased such that 5% of infants are below the 3^rd^ centile and 5% above the 97^th^.

## 1 ANDERSON, HAFEN, SOFRYGIN, RYAN, AND HBGDki COMMUNITY

The two papers from Dr Anderson and colleagues are among the first published outputs from the Healthy Birth Growth and Development knowledge integration (HBGDki) initiative, a major project funded by the Gates Foundation.^1^ Its aim is to reduce the global burden associated with poor nutritional status and impaired cognitive development in young children, by assembling data on growth and development from nutrition studies worldwide and by meta‐analysing the individual data.

The aim of the first paper is to describe and compare modern methods for analysing longitudinal growth trajectories of length/height and weight, with a view to making recommendations for future practice. The scale of the initiative is impressive—the project has so far collected data on over 100 studies, and the first paper here focuses on 21 studies involving over 120 000 children and almost 800 000 measurement occasions. (In parenthesis, one difficulty with the project lies in its name—the acronym HBGDki is unmemorable and easily confused with the deceptively similar HBDGki, which also appears several times in their paper.)

### 1.1 Growth trajectory models

Six growth trajectory models are considered: linear and quadratic versions of Laird‐Ware,^2^ my own SITAR,^3^ brokenstick,^4^ multilevel spline,^5^ and functional PCA or FACE.^6^ All the analysis is done in *R*, with the code available in the *hbgd* package,^7^ a very powerful software resource. To explore the findings in greater depth, it is useful to understand in broad terms how the growth models differ. They are all mixed models, with random subject effects, and as the authors say “there exists an overall mean curve for a particular population, and the differences between children can be explained as deviations from this mean curve.” The models differ in terms of the smoothness of the mean curve and also the smoothness of individual deviations (departures) from the mean curve. Smoothness here is quantified in terms of *degrees of freedom* or d.f., reflecting the order of a polynomial or the number of knots in a spline curve.

The d.f. for the Laird‐Ware models are two (linear) and three (quadratic) for the mean curve and, similarly, two and three for each subject's deviations. The d.f. for the SITAR mean spline curve are user‐selected, though the authors do not state what they used. Then, each subject has three d.f. to summarise departures from the mean curve, reflecting transformations of the mean curve corresponding to shifts on the measurement and age axes, plus a scaling of the age axis. Thus, in this sense, SITAR and quadratic Laird‐Ware are similar, each having three d.f. for deviations and around three d.f. for the mean curve (depending on the exact choice for SITAR).

The remaining three models all involve specifying a number of knots for spline curves (the brokenstick is a linear spline model, while FACE fits cubic splines), and in this analysis, the software defaults were used, though again, we are not told what the defaults were nor how they were determined. Among other things, the required number of d.f. will depend on the size and age range of the data, which extended from birth to 6954 days or 19 years in the most longstanding studies (Table 1). A height trajectory from birth to 19 years is complex in shape and requires at least eight d.f. to fit adequately, but only two studies lasted this long and the median upper age across studies was 3 years; for such early life studies, the curve shape is much simpler, requiring only three or four d.f.

### 1.2 Comparing model trajectories

The models are first applied to the Content dataset (children aged 0 to 2 years from a low income country), and the results are summarised in Figures 1 and 2, showing the data and fitted curves for 11 distinct growth models as applied to “a single randomly selected child” (quotation marks mine—see later). Figure 1 contains results for the six models applied to height, while Figure 2 has the equivalent results for five models with height Z‐score (HAZ), where SITAR is omitted as it lacks biological sense on the Z‐score scale.

The differences between models in Figure 1 are quite subtle, with the exception of linear Laird‐Ware where the fitted curve is a straight line. Quadratic Laird‐Ware is very similar to SITAR, and both fit reasonably well; this implies that both are using three d.f. for the mean curve and three for subject deviations. However, the authors feel that the Laird‐Ware models “are not flexible enough to model a sensible growth curve,” while SITAR and the other three models “do a reasonable job.”

The brokenstick and FACE curves are appreciably less smooth than the other four, reflecting more d.f. to play with. Each curve represents the sum of the model mean curve and the subject deviations, so the extra d.f. may be allocated to either or both. Figure 1 does not give the mean curves, so it is not possible to tell how smooth they are.

Figure 2 shows the analogous results on the HAZ scale. The multiple correlation is much smaller than in Figure 1, and the plots considerably noisier, because the age trend in height has largely been removed by the Z‐score transformation. When applied to the WHO reference sample, the mean Z‐score curve is given by *HAZ*(*t*) = 0 in an obvious notation, a horizontal straight line through the origin, so the mean curve is the simplest possible. In practice, the study mean curves will not be so simple, but HAZ in LME countries is known to fall during the first year and then plateau, a much simpler pattern than seen in Figure 1. Thus, the models in Figure 2 ought to allocate fewer d.f. to the mean curve than to the subject deviations, though again, there is no information on this. The authors acknowledge (on pages 4 and 9) that fewer d.f. are needed on the Z‐score scale, but it is a shame that they do not expand on the statement.

What is curious about Figure 2 is the child's trajectory; HAZ increases monotonically with age, from −1.5 at birth to +1.0 at 2 years, ie dramatic length gain, in stark contrast to the early fall then plateau that one would expect for a low income country. It is hard to believe that the child was randomly selected, though equally, it is puzzling why such an extreme trajectory might have been chosen.

The two Laird‐Ware models perform poorly in Figure 2, ie, they are too smooth (inflexible) to pick up short‐term variation in HAZ. The other three models are less smooth and broadly similar in terms of flexibility. With the increased detail, it is possible to see that the brokenstick curve consists of five segments, ie four d.f., and the two spline model curves are similar in shape and probably have a similar number of d.f. (though smoothed with cubic rather than linear splines).

### 1.3 Comparing model fit

On the back of this analysis, the authors decide to focus on the penalised spline, brokenstick, and FACE models. The Laird‐Ware models are dismissed owing to their poor fit, while SITAR is overlooked because its convergence can be a problem with larger datasets, and it does not fit naturally on the Z‐score scale. The cross‐validation analysis for the three remaining models is extended to all 21 studies, and the results are shown in Tables 2 and 3.

The mean squared errors (MSEs) vary considerably from study to study, probably reflecting differences in age range. FACE fails to fit for 8 of the 21 studies, and MSEs are smaller on the Z‐score scale than the height scale, where brokenstick and FACE perform best. Figures 3 and 4 confirm that brokenstick fits better on the Z‐score scale, though the figures would work better with the MSE axes on log scales.

The better fit on the Z‐score scale is probably due to the extra d.f. available for subject deviations, given that the mean curve is simpler and the models all use the default d.f. Tables 4 to 6 explore this by varying the number of knots in the three models applied to dataset E (presumably Content, and presumably on the Z‐score scale). What they demonstrate, at least for brokenstick and FACE, is a simple truth—using more d.f. for subject deviations improves the fit.

However, it is striking that unlike brokenstick and penalised spline, FACE performs very similarly on the height and Z‐score scales. This is surprising, and it challenges the authors' recommendation to always work on the Z‐score scale.

Given the massive amount of work involved in assembling Tables 2 and 3, it would be interesting to explore them in more detail by predicting the MSE in terms of study‐specific factors such as the numbers of children and observations, distribution of time gaps, age range, etc. This would, for example, evidence the authors' statement that brokenstick performs better with fewer observations per child.

### 1.4 Growth faltering, time interval, and smoothing

Overall, the authors conclude that brokenstick and FACE are useful and work best on the Z‐score scale. But, this leads to the bigger question: how should the results of the analyses be exploited?

The aim of characterising growth trajectories is to relate growth faltering to later outcome. The authors suggest three examples of relevant growth indicators based on the trajectories: “mean growth over a particular time period, number of days in a particular growth state, or indicators relating to growth derivative.” They all involve specifying a time period, which can be short or long. For example, they particularly mention the mean derivative, ie, the increment, over the first year.

This dependence on the time scale is fundamental to growth assessment, and it relates directly to the smoothness of the growth trajectories. For example, the brokenstick curve in Figure 2 has five segments spread over 700 days, each segment corresponding on average to 140 days or 4.6 months. This is the timescale on which growth faltering will be detected: on a shorter timescale, faltering is smoothed out, while on a longer timescale, the noise obscures the growth signal. This means that the brokenstick model is tuned to growth assessment over this period of  ∼5 months. Conversely, the quadratic Laird‐Ware or SITAR curves are smooth across the whole age range and hence apply on that timescale, which could be 2 years or longer.

The example given of growth increment over the first year is instructive. FACE and brokenstick are tuned to shorter timescales than this, so they may not predict first year increment any better than quadratic Laird‐Ware or SITAR. If the analysis were to be repeated using just the first year data, then quadratic Laird‐Ware or SITAR would provide exactly the required individual growth summary—an intercept corresponding to their length, and a slope corresponding to their length increment. In addition, SITAR is unique in providing an estimate of the individual's age at peak length velocity, which occurs in the first month or two after birth and which is a useful summary statistic of early growth intensity that the other models lack.

### 1.5 Conclusion

In summary, I am impressed by the colossal amount of work that has been put into the project. The recommendations to use brokenstick or FACE on the Z‐score scale are reasonable and well evidenced, demonstrating that the two models quantify growth faltering on a timescale of a few months. Against that I am slightly disappointed that the results are not more extensive, given the vast amount of data available.

But the key issue, which the paper does not address, is whether such a timescale is optimal for predicting later adverse outcome. It is assumed that, since brokenstick and FACE explain the most variability, then the timescale on which they operate must be optimal for prediction. But this does not follow at all; short‐term HAZ variability may be less relevant than long‐term variability for predicting later health outcome. It is important that the models be compared, not only on their ability to predict early growth but also on their ability to predict later outcome. Such a comparison will need to include quadratic Laird‐Ware and SITAR, as they provide parsimonious and unambiguous summaries of early growth in individuals that the more complex brokenstick and FACE do not and cannot provide.

## 2 ANDERSON, XIAO, CHECKLEY, AND HBGDki COMMUNITY

The second of the two Anderson papers aims to simplify the comparison of measures of weight or height at two ages in the same individual. The rate of change in size over time represents growth velocity, and the child's expected velocity depends on their two ages of measurement. To quantify the expected velocity requires a growth velocity reference, analogous to the fetal size references of Ohuma and Altman (see later). However, velocity references are complex to construct because each velocity measurement involves two ages not one, and hence two measurement errors, which interact with the time interval between measurements in a complex way.

An alternative approach, which avoids the need for a velocity reference, is to switch from the measurement scale (ie, kg or cm) to the Z‐score scale. The Z‐score (or standard deviation score) is the measurement transformed to a normal equivalent deviate, so that when applied to the reference population, it has mean 0 and SD 1 irrespective of age or sex. Anderson et al use the World Health Organization growth standard as reference.^8^

The properties of Z‐scores make it much easier to compare two measurements in an individual^9^ and lead to Equation (3) of Anderson et al that measures growth velocity conditional on the first of the two measurements, or conditional velocity for short. The equation involves the two Z‐scores and the correlation between them, ie, the correlation of measurements obtained at the two measurement ages. In general, this correlation is not known, and the aim of the paper is to provide a meta‐analytic estimate of the correlation for a two‐way grid of measurement ages. In this way, it becomes possible to calculate conditional velocity for any pair of Z‐scores simply by looking up the correlation for the two measurement ages.

There is, however, a tension between individual ages and age groups. The age scale needs to be discretised to provide groups of measurements, which can then be used to calculate the correlations across age groups. But, these correlations then relate to the age group, not a specific age. Anderson et al address this by analysing the data in weekly age groups (*n* = 940, ie, from birth to 18 years), which provides useful granularity close to birth but considerable redundancy at older ages. Note that 940 cells^2^ for the correlation matrix is a large number, and many of the cells will have few or no data. So, it would have been interesting to explore reducing the number of age groups by letting them vary in width, while preserving the detail of the correlation structure. An even neater approach would be to develop a way to calculate the correlations based on individual ages rather than age groups.

To combine the correlation matrices across studies, the authors consider two alternative approaches, ie, univariate and multivariate. The univariate approach combines studies by applying a meta‐analysis to study correlations for each cell of the correlation matrix and then smooths the resulting matrix, while the multivariate approach smooths each study's correlation matrix as a surface and then combines the surfaces. In practice, the univariate approach turns out to be easier. The meta‐analysis works with Fisher‐transformed correlations, and it estimates study‐specific random effects assuming that all the cells of each study's correlation matrix are larger or smaller than the mean by the same amount. In practice, this may be unrealistic because studies will have different measurement error structures at different ages, with some studies having noisier measurements (and hence lower correlations) in infancy due to say an adverse environment; but, the same may not apply at later ages where growth patterns are less susceptible to environmental pressures. To what extent taking this into account might affect the correlation matrix is unclear.

The heat maps showing the age‐varying correlation structure are impressive. In general, they show that correlations for measurements close in age are high, but that the correlations fall as the time gap increases. Also, the rate at which the correlation falls with increasing time gap is greater in infancy than later in childhood. What the heat maps do not show is the value of the correlation near the diagonal at different ages; this is of interest as its difference from unity reflects the measurement error at that age (or nugget). The smoothed heat maps in Figures 4 and 5 show several circular patches of dark red along the diagonal, indicating peaks in the correlation, and it would be interesting to know if they reflect biological landmarks.

One practical problem with the heat maps is that they are not transferable. Researchers wanting to use them to calculate growth velocity do not have access to them, so that making the information available online will be important.

## 3 OHUMA AND ALTMAN 1

The two papers by Dr Ohuma and Professor Altman describe the statistical methodology behind the fetal and neonatal growth references produced by the INTERGROWTH‐21^st^ project, funded (like the HBGDki) by the Gates Foundation. Similarly to Anderson and colleagues, they focus primarily on growth in early life, but unlike Anderson et al, who assess individual postnatal growth, they quantify the distribution of normal fetal and neonatal size and growth by gestation.

Their first paper reviews design considerations for such references, in terms of data collection, handling, and analysis, while the second describes the statistical analysis for two of the references presented as worked examples. I refer to the papers as OA1 and OA2, respectively.

The design review in OA1 is comprehensive and highlights deficiencies in current practice and includes recommendations for improvement. To quote the Abstract, “Important design features such as inclusion and exclusion criteria, ultrasound quality control measures, sample size determination, anthropometric evaluation, gestational age (GA) estimation, assessment of outliers, and chart presentation are seldom well addressed, considered or reported.” Those familiar with Altman's work will recognise the tone, reflecting his longstanding concern with poor reporting practice. Below, I respond to some of the points raised, grouped using section headings taken from their review.

The key feature of the INTERGROWTH‐21^st^ project is that it provides prescriptive rather than descriptive growth references, based on reference samples of fetuses and infants from eight countries selected to be “healthy.” This matches the design of the World Health Organization growth standard,^8^ which was based on infants and young children from six countries born at term and selected for health. However, the concept of “health” is harder to apply to infants born preterm than to those born at term, since being born preterm is clearly not healthy. INTERGROWTH‐21^st^ draws a distinction between healthy and unhealthy preterm infants by applying carefully chosen selection criteria.

### 3.1 Cross‐sectional, longitudinal, or mixed study designs

Study design for growth references relates to the nature of the data, cross‐sectional or longitudinal, ie, whether individuals are measured once or repeatedly. The former leads to charts of size, ie conventional growth centile charts, while the latter assesses growth velocity. Ohuma and Altman point out that, in practice, longitudinal data are often used to construct size charts, by ignoring the repeated measures in the data. This is effective though inefficient, as the centiles are, in general, unbiased but less precise; repeat measures are unbiased so long as they are independent of the way they are collected.^10^

Linked to this, the authors also draw a clear distinction between size charts and growth charts, the phrase “size and growth” appearing several times in the paper. However, they do not make clear that growth charts monitor change in size over time in the form of growth *velocity* charts, which are quite different in appearance from size charts. They discuss growth velocity charts hardly at all.

### 3.2 Multiple centres

An important design issue that arises for a multicentre study such as INTERGROWTH‐21^st^ is whether or not the data from particular centres can validly be included. INTERGROWTH‐21^st^ used data from multiple countries to obtain an international growth standard, though the countries inevitably differed in detail from each other. The advantage of a *prescriptive* international standard is that growth statistics based on the standard can be compared directly across countries, whereas with locally representative *descriptive* growth references such comparisons cannot be made. Conversely, local descriptive references can—so long as they are up‐to‐date—provide unbiased assessments of centile position in individual children, because the centiles are representative of the target population, and an international prescriptive reference cannot do this. Thus, there are pros and cons with both approaches.

### 3.3 Quality control

The paper's focus on fetal and neonatal charts makes the assessment of gestational age particularly important. Ohuma and Altman describe how best to estimate gestational age, using the last menstrual period combined with the ultrasound dating scan. They emphasise the need for quality control, checking for variability both between and within ultrasonographers, in the same way as for anthropometry in postnatal growth studies. To control for variability, the INTERGROWTH‐21^st^ study scans were all carried out in triplicate, which could then be averaged or modelled in the fitting process.

Incidentally, another way to summarise the triplicate measurements would have been with the median rather than the mean, as it is more robust if the error distribution has heavier tails than normal.^11^ The median also corresponds directly to one of the triplicate measurements, and it needs no calculation.

Later, OA1 discusses the issue of single versus repeated measurements in terms of variability. Using the mean or median of replicate measurements reduces measurement error, but it can also introduce bias. The issue is how the centiles are used in clinical practice—if single rather than repeated measurements are routinely used, then the data will be noisier than the centiles reflect.

Linked to quality control is the question of data management—the identification and handling of data outliers. OA1 mentions outliers in passing, but without any discussion about data cleaning. This is the important first stage in the statistical analysis process, and it needs to be formalised in the protocol.

### 3.4 Sample size

The topic of sample size for growth studies is surprisingly complicated, for both size and growth charts, as the authors note. I discussed the topic at length in 2006 and it appears to have hardly advanced since then.^12^ If the sample size calculation is based on the precision of the extreme chart centiles, as is logical, it is not obvious how precise they need to be nor how the precision should depend on gestational age or the degree of smoothing. A particular difficulty with neonatal size references is their dependence on gestational age, such that the vast majority of infants are born at term (37 to 42 weeks), while the numbers born preterm become progressively smaller with increasing prematurity. Sample size considerations for growth velocity charts are further complicated by the need to incorporate the correlation between successive measurements.

The INTERGROWTH‐21^st^ birthweight reference was based on 20 302 neonates,^13^ but 19 280 of them were born at term (OA2, Table 1). Preterm births were poorly represented, particularly at the earliest gestations, and there were just 113 and 51 infants, respectively, at 34 and 33 weeks gestation (33 weeks being the study's start gestation). These numbers are clearly very small to model centiles adequately, particularly when split by sex. The lack of data for extremely preterm neonates born before 33 weeks gestation is another weakness, since birthweight assessment in the extremely preterm is particularly important for management.

This fact was belatedly acknowledged by the later publication of INTERGROWTH‐21^st^ centiles for very preterm neonates,^14^ based on 408 infants from 24 to 32 weeks gestation, although they included the health warning that “centiles below 28 weeks should be interpreted with caution given the small sample size.”^15^

For comparison, the England and Wales birthweight centiles from 24 to 42 weeks gestation recently published by Norris et al^16^ in were based on 1.2 million births, of which 4954 were born before 32 weeks. With numbers like these, one has confidence in the accuracy and precision of the extreme centiles at the earliest gestations, which, to be honest, one does not with the INTERGROWTH‐21^st^ centiles.

### 3.5 Routinely collected versus research data

The key difference between the Norris paper and INTERGROWTH‐21^st^ is the use of routinely collected rather than research data. OA1 discusses the pros and cons of using routine data and concludes that, with certain caveats, they can be a valuable resource. They also note that routinely collected birthweight data are generally accurate, which raises the question as to why INTERGROWTH‐21^st^ did not make use of them to increase their sample size.

### 3.6 Statistical methodology

Ohuma and Altman's (OA1) main requirements for the statistical construction of growth centiles are that they should change smoothly with gestation, provide a good fit to the data, rely on the simplest statistical model necessary, and allow calculation of Z‐scores. The chosen model should test for goodness of fit, including the normality assumption, and estimate both mean and standard deviation (SD) as functions of gestation. In addition, they expect to see reported a raw data summary and the fitted centiles by gestation, a graphical comparison of fitted centiles superimposed on the raw data, and regression equations for the mean and SD.

## 4 OHUMA AND ALTMAN 2

The second paper by Ohuma and Altman provides two worked examples of centile construction, chosen to illustrate the different methodologies for cross‐sectional and longitudinal data. The first covers birthweight and the second fetal head circumference.

### 4.1 Birthweight centiles

When constructing birthweight centiles, the first stage of the analysis is to choose the regression model. The aim is to estimate the gestation‐varying distribution of birthweight in terms of smooth gestation‐specific curves for the distribution moments: the mean, SD, and possibly skewness and kurtosis. Then, if the moment curves are smooth, the centiles curves based on them will be smooth too.

Ohuma and Altman (OA2) consider fractional polynomials, first described by Royston and Altman;^17^ the LMS method of Cole and Green;^18^ and two extensions of LMS, the LMSP^19^ and LMST^20^ methods of Rigby and Stasinopoulos. All these models are members of the GAMLSS family (generalised additive models for location, scale, and shape),^21^ which can be fitted using GAMLSS software.^22^

GAMLSS specifies each model in terms of its frequency distribution and the smoothing technique used. Commonly used distributions (there are over 70 to choose from!) include the normal (NO), Box‐Cox Cole‐Green (BCCG, as used by the LMS method), and the LMS extensions Box‐Cox power exponential (BCPE) and Box‐Cox *t* (BCT) as used by LMSP and LMST. The models differ in terms both of how many distribution moments they have (respectively 2, 3, 4, and 4 above) and of the simpler distribution they are based on (respectively normal, normal, power exponential, and *t*). So, LMS, LMSP, and LMST all model skewness, and in addition, LMSP and LMST model kurtosis. OA2 also use three other distributions, power exponential (PE), skew power exponential type 3 (SEP3), and skew *t* type 3 (ST3), where PE models kurtosis while SEP3 and ST3 model skewness and kurtosis.

Like the distribution family, the specification of the smoothing technique in GAMLSS is enormously flexible. Common choices are a polynomial (eg linear or quadratic), or a fractional polynomial, or one of the many forms of spline curve. The basic OA2 fractional polynomial model is actually a fractional polynomial with a normal distribution, while the LMS method as originally described was a cubic smoothing spline with a BCCG distribution. Similarly, LMSP and LMST are typically fitted using cubic splines. Figure 2 of OA2 illustrates the GAMLSS taxonomy, though it mixes up fractional polynomials—a smoothing technique—with LMS models that represent distinct distributions.

After this preamble, we now look at the birthweight centiles analysis in Table 2 of OA2. There are 10 models for each sex, which can be most simply compared via the AIC and BIC. Both criteria fall with rising model complexity, and the values for LMS, LMST, and LMSP are all smaller than for the corresponding fractional polynomial models in both sexes (*the BIC of 62 for SEP3 in girls should presumably be 162*). This indicates that cubic splines are better than fractional polynomials for fitting the moment curves.

The best fractional polynomial fits are with the ST3 distribution, and Ohuma and Altman select this as their “best buy”, on the basis of centile appearance and linearity of the worm plots and Q‐Q plots (note that worm and Q‐Q plots are identical apart from a 45° rotation). This is surprising, given that the fractional polynomials fit worse by AIC and BIC, and it is a pity that the authors provide no evidence to support the statement that the fit is better in terms of centiles and residuals.

Figure 3 compares three predicted centiles (*3* ^*rd*^ *50* ^*th*^ *97* ^*th*^ *?*) from four models ( *for boys?*)—fractional polynomials with NO, BCCG, and ST3, and cubic splines with BCCG. It is striking that the centiles for the four models agree closely for 36 to 41 weeks gestation, and the material differences between them are restricted to 33 and 34 weeks, where the data are sparse. The fact that elsewhere the model centiles match well indicates that the differences between the models are nothing to do with the distribution, and all to do with the curve shape—ie, the smoothing technique—at the extremes.

But, there is a further wrinkle. Ohuma and Altman state repeatedly that the chosen model should be as simple as possible, and Figure 3 shows there is little to choose between four of them. Six of the 10 models in Table 2 adjust for kurtosis, yet there is no sign in Figure 3 that the kurtosis adjustment makes any difference—the NO (mean and SD) and ST3 (mean, SD, skewness, and kurtosis) centiles are almost identical.

Kurtosis reflects heavy or light tails to the distribution, as shown by the S‐shape in the worm plots of Figures 4 and 5. For the normal distribution (Figure 4), the worm is near linear between −2 and +1.5, whereas for the ST3 distribution (Figure 5A), the linearity extends from −3 to +3, clearly far better. So, if kurtosis is so much better with ST3, why does not it show in Figure 3? The reason is that the centiles there are not extreme enough to show it; ±3 corresponds to the 0.1^th^ and 99.9^th^ centiles, so centiles that are less extreme than these are unaffected by kurtosis. The outer centiles on the INTERGROWTH‐21^st^ charts are the 3^rd^ and 97^th^, which are far less extreme than the 0.1^th^ and 99.9^th^; for this reason, there is no point in modelling kurtosis—a simpler model with mean, SD, and skewness would perform just as well.

This example highlights the care needed to model with GAMLSS. The plethora of available models and smoothing techniques can seduce the analyst into trying them all and selecting the best. But, as we have seen, the six kurtosis models could have been ruled out at an early stage, since the kurtosis adjustment was never going to affect the centiles.

Another general modelling principle would be to exploit the data structure by “borrowing strength”. The two sexes are modelled separately here, yet it is known that their distributions are very similar, with girls slightly lighter than boys on average at all gestations. Thus, fitting a model with the sexes combined and including a sex effect would simplify the model to advantage, particularly at the early gestations with few data. This was the approach used to model birthweight in very preterm UK infants,^23^ fitting the LMS method with a log link for μ, where the mean sex difference in birthweight was 6.6%.

### 4.2 Fetal head circumference centiles

Fetal head circumference in INTERGROWTH‐21^st^ was measured longitudinally during pregnancy,^24^ with a mode of 5 measurements per subject (range 1 to 6) (OA2 Table 3). Thus, the data structure of measurements at level 1 and subjects at level 2 implies a hierarchical (multilevel) model. In addition, each measurement was made in triplicate (actually, three separate images were each read three times^24^), which can be viewed as an extra level 0. Ohuma and Altman fit four alternative hierarchical models reflecting this structure, as summarised in OA2 Table 4.

They assume a normal distribution for all and model the mean and SD with fractional polynomials. Based on the deviance, there is evidence of heterogeneity between subjects in the intercept and slope, but the most interesting columns in the table are the percentages of observations outside the 3^rd^ and 97^th^ centiles. Nominally these should be 3%, and the values for the simple random intercept model are close to 3%, but with increasing model complexity, this tail area increases, reaching 5% with the three‐level model.

Somewhat surprisingly, this latter model is the one chosen as optimal. It is a surprising choice because the model is severely biased in the tails, with 5% of observations below the 3^rd^ centile and 5% above the 97^th^ centile. Neonatalogists expect to see 3% not 5% of infants in the tails and are likely to be both surprised and confused to know there are 67% more than expected.

What if anything could have been done to avoid this bias? The seminal paper by Royston^25^ showed how to model longitudinal fetal data to produce unbiased centiles, and his method could be applied here. It involved transforming both the measurement scale and the gestational age scale so as to make the mean curve linear, which could then be modelled with random intercepts and optionally random slopes. In this case, the variance is a function of the level‐1 and level‐2 variances. Because the mean curve is linear the subject random effects do not introduce bias in the centiles.

## 5 CONCLUSION

The statistical analysis of the INTERGROWTH‐21^st^ reflects a major undertaking by the authors. However, I feel that the outcome could have been better with a more nuanced approach to the analysis and with a greater awareness of how the resulting charts are used in clinical practice.
